# Vermicompost promotes apple seedling growth by enhancing photosynthetic efficiency and driving chlorophyll biosynthesis

**DOI:** 10.3389/fpls.2026.1904356

**Published:** 2026-09-03

**Authors:** Qi Zhao, Huizhen Wang, Huaping Yang, Haoshuai Li, Yanan Chen, Dehui Zhang, Yongbo He

**Affiliations:** College of Horticulture, Shanxi Key Laboratory of Protected Horticulture, Shanxi Agricultural University, Taiyuan, China

**Keywords:** apple seedling, chlorophyll biosynthesis, growing substrate, photosynthetic efficiency, physicochemical properties, vermicompost

## Abstract

To evaluate the comprehensive effects of different vermicompost incorporation ratios on the growth performance, nutrient accumulation, and photosynthetic efficiency of apple seedlings (G935, Geneva 935), plants were treated with varying proportions of vermicompost (0%, 50%, 80%, and 100%). Results indicated that increasing vermicompost proportions significantly optimized growing substrate physicochemical properties. Specifically, growing substrate pH decreased from a baseline of 8.02 to 7.78 under 100% vermicompost treatment, a modest reduction that crucially improves micronutrient solubility (especially Fe and Zn) in alkaline conditions, while electrical conductivity (EC), organic carbon, and total nutrients were substantially elevated from their respective control levels. Additionally, vermicompost robustly promoted apple seedling growth and significantly enhanced light energy capture efficiency by increasing the maximum photochemical efficiency of photosystem II (F_v_/F_m_), actual photochemical quantum yield [Y(II)], electron transport rate (ETR), and non-photochemical quenching (NPQ). The 100% vermicompost treatment maximized these benefits, driving total chlorophyll content and leaf nitrogen concentrations from 4.25 mg g^-1^ and 7.71 mg g^-1^ in the control to 9.37 mg g^-1^ and 12.34 mg g^-1^, respectively. Mechanistically, this enhanced light utilization was driven by the significant accumulation of chlorophyll biosynthesis precursors—including 5-aminolevulinic acid (ALA), magnesium protoporphyrin IX (Mg-Proto IX), and protochlorophyllide (Pchlide)—relative to their control baselines (e.g., ALA increased from 15.98 ng·g^-1^ FW to 22.47 ng·g^-1^ FW). In conclusion, vermicompost improves growing substrate properties, enhances nutrient supply, and specifically stimulates the chlorophyll biosynthetic pathway, thereby increasing light energy utilization efficiency and providing solid theoretical support for its application in fruit tree nursery production.

## Introduction

Apples (*Malus domestica* Borkh.) are economically vital fruit crops in the global horticultural industry (Kumar et al., 2024). The successful establishment of modern high-density orchards relies heavily on the production of high-quality, vigorous nursery seedlings. In containerized seedling production, the physicochemical properties of the growing substrate are critical determinants of early vegetative growth and physiological performance (Kim et al., 2021). However, conventional nursery practices often rely on synthetic fertilizers or non-renewable peat, which can restrict macronutrient bioavailability and limit the photosynthetic potential of grafted seedlings (Zhao et al., 2023; Zhuang et al., 2024). Therefore, developing ecologically sustainable, high-quality nursery substrates is urgently required to optimize the microenvironment and stimulate the rapid physiological development of apple saplings.

Organic soil amendments, particularly vermicompost, offer an effective eco-friendly solution for transplant seedling production, as compost has been widely verified as a reliable peat substitute for nursery substrates (Jahromi and Aboutalebi, 2011; Rohim et al., 2024; Li et al., 2026). Produced via non-thermophilic biodegradation by earthworms and microbes, vermicompost features a highly porous matrix and excellent water retention (Safadoust et al., 2025). It actively buffers soil pH and efficiently mineralizes complex organic materials into readily plant-available nutrients, such as nitrate nitrogen, available phosphorus, and potassium (Reis et al., 2025). These profound microenvironmental improvements translate directly into robust plant biomass and root development (Ahmad et al., 2024; Manzoor et al., 2024). For example, in the nursery cultivation of ‘Fuji’ apple and sweet cherry (*Prunus avium*) saplings, vermicompost application has been proven to significantly alleviate substrate compaction, driving the proliferation of fine feeder roots and increasing overall root surface area for superior nutrient interception (Yüca and Pirlak, 2022).

Numerous studies have demonstrated that vermicompost application can obviously accelerate plant growth and increase biomass accumulation (Bai et al., 2025; Tüzel et al., 2025; Abiz et al., 2026). This robust vegetative growth is inextricably linked to enhanced photosynthetic capacity, primarily driven by Photosystem II (PSII) (Chen et al., 2020). Chlorophyll fluorescence analysis—utilizing key parameters like maximum photochemical efficiency (F_v_/F_m_), actual quantum yield [Y(II)], and non-photochemical quenching (NPQ)—has become an indispensable tool for evaluating light energy utilization (Jiao and Hu, 2025; Hamerlynck and O’Connor, 2026). Previous studies on crops like potted grapevines, citrus orchards, and field-grown maize demonstrate that high-quality organic amendments can upregulate photochemical traits by optimizing CO_2_ assimilation (Maia et al., 2026; Xu et al., 2024; Wan et al., 2021).Specifically in tree fruits, organic soil conditioning has been shown to sustain high F_v_/F_m_ ratios in young apple trees subjected to nutritional stress, preventing the rapid degradation of PSII reaction centers (Ferreira et al., 2026; Pang et al., 2026). The biochemical basis for this elevated photosynthetic efficiency lies in the abundant foliar accumulation of photosynthetic pigments, governed by the chlorophyll metabolic pathway (Lokstein et al., 2021). Biosynthesis begins with 5-aminolevulinic acid (ALA) and progresses through intermediates like protoporphyrin IX to form mature chlorophyll (Wu et al., 2019). This cascade is highly dependent on an adequate supply of specific mineral nutrients: nitrogen (N) is a core component of the tetrapyrrole ring, magnesium is the central coordinating atom, and iron (Fe) acts as an indispensable catalytic cofactor (Kroh and Pilon, 2020; Sinha et al., 2022). For instance, Fe deficiency in high-pH calcareous orchard soils frequently induces severe interveinal chlorosis in emerging apple leaves due to the abrupt halt of ALA synthesis (Fu et al., 2015). Notably, vermicompost application can slightly decrease soil pH in calcareous soils through earthworm-secreted organic acids and organic matter decomposition. This pH-lowering effect promotes the dissolution of insoluble iron oxides, converts fixed Fe into plant-available fractions, and thus mitigates Fe deficiency stress driven by high soil pH. The natural chelating properties of vermicompost, however, can maintain these essential trace metals in plant-available forms, thereby sustaining the pigment biosynthetic cascade (Gholami et al., 2019; Talaat and Abdel-Salam, 2024). Yet, whether vermicompost drives total chlorophyll accumulation by actively upregulating intermediate precursors or merely suppressing the degradation pathway remains uncharacterized in apple nursery production. To address this unresolved mechanistic gap, we quantified both key chlorophyll biosynthetic precursors and representative degradation products (pheophytin a and chlorophyllide a) in this study, to distinguish whether vermicompost modulates chlorophyll metabolism primarily by enhancing precursor biosynthesis or by suppressing the degradation process.

To elucidate these interconnected regulatory mechanisms, we designed a controlled experiment utilizing G935 apple rootstock seedlings. G935 was specifically selected due to its widespread adoption in modern high-density orchards, where its semi-dwarfing vigor and high yield efficiency make early nursery establishment and optimal nutrient management practically crucial. These seedlings treated with varying vermicompost incorporation ratios (0%, 50%, 80%, and 100%). We quantified resulting modifications to growing substrate physicochemical properties, morphological plant growth, and detailed chlorophyll fluorescence parameters. Most importantly, to dissect the molecular-physiological basis of improved photosynthesis, we profiled intermediate precursors, key degradation products, and foliar mineral accumulation (N, Mg, Fe) within the chlorophyll pathway. We hypothesized that vermicompost promotes chlorophyll accumulation primarily by enhancing mineral availability and actively driving intermediate precursor synthesis, rather than simply inhibiting degradation processes. The empirical findings aim to provide a theoretical foundation for the sustainable application of vermicompost in modern fruit tree nursery systems.

## Materials and methods

### Materials and experimental design

In May 2024, tissue-cultured G935 apple seedlings with uniform growth were selected and transplanted into cylindrical seedling cups. The cups were composed of bentonite and straw at a mass ratio of 5:5, with an outer diameter of 10 cm, a height of 20 cm, and a wall thickness of 1 cm. Before planting, the vermicompost and growing substrate were thoroughly mixed according to the designated ratios (by volume). Four treatment groups were established: CK (100% growing substrate), V5 (50% growing substrate + 50% vermicompost), V8 (20% growing substrate + 80% vermicompost), and V10 (0% growing substrate + 100% vermicompost). Vermicompost was produced by Shanxi Wanmu Feng Fertilizer Co., Ltd (Jinzhong, China) following our optimized process through earthworm digestion of composted cattle manure. For each treatment, 30 uniformly growing plants were used, and field management was consistently maintained throughout the experiment. The trial was conducted in a greenhouse in the Xiaodian District, Taiyuan City, Shanxi Province (37°82′N, 112°56′E). The treatment period lasted for 20 d.

### Ambient greenhouse conditions

To ensure experimental reliability, all treatment and control groups were cultivated simultaneously on the same greenhouse bench, experiencing the exact same environmental fluctuations. Furthermore, all chlorophyll fluorescence measurements were conducted following strict standard protocols (e.g., 30-minute dark adaptation) to minimize transient environmental noise.

### Determination of growing substrate physicochemical properties

Growing substrate samples were collected from five randomly selected apple seedlings of each group and mixed as one replicate. The samples were air-dried, ground, and passed through a 2-mm sieve for subsequent analysis. The pH and electrical conductivity (EC) of growing substrate were determined with a pH meter (PHS-3C, Leici, Shanghai, China) and a conductivity meter (DDS-370, Leici, Shanghai, China), respectively (Mosley et al., 2024), using substrate suspensions prepared at a solid-to-water mass ratio of 1:2.5. The specific testing methods for growing substrate nutrients were listed below: total organic carbon (TOC) was measured by sulfuric acid digestion-potassium dichromate external heating method; total nitrogen (TN) was determined by the Kjeldahl digestion method; total phosphorus (TP) was analyzed using vanadium-molybdenum yellow colorimetry; total potassium (TK) underwent H_2_SO_4_-H_2_O_2_ digestion before flame photometric measurement; available phosphorus (AP) was extracted by sodium bicarbonate solution and quantified through molybdenum-antimony anti-colorimetry; available potassium (AK) was extracted with ammonium acetate solution and detected via flame photometry (Li et al., 2024). Nitrate nitrogen (NO_3_^-^-N) was extracted with 1 mol L^-^¹ KCl solution and assayed with a continuous flow analyzer (Lei et al., 2026). All growing substrate physicochemical and nutrient determination experiments were performed with three biological replicates.

### Plant growth measurements

After 20 d of planting, for each treatment, five seedlings were randomly selected and carefully excavated, washed three times with tap water, followed by rinsed three times with deionized water. Plant height was measured from the stem base to the apical bud using a measuring tape (precision: 0.1 cm), and stem diameter was measured at the stem base using a vernier caliper (precision: 0.01 mm). Then the plant seedlings were separated into roots, stems, and leave and each organ was sealed in a paper bag. Samples were heated at 105 °C for 30 min for enzyme inactivation, then dried at 65 °C until constant weight. Organ dry weight (DW) was measured by an electronic balance (0.001 g precision). Plant biomass was calculated as the sum of roots, stems, and leave DW. Net biomass accumulation was calculated as follows: Net biomass = *DW*_2_ (at day 20) - *DW*_1_ (at day 0). The relative growth rate (RGR) was calculated using the following: RGR = (ln *DW*_2_ - ln *DW*_1_)/(*t*_2_ - *t*_1_) (Liang et al., 2018). The strong seedling index was calculated as follows: strong seedling index = (stem diameter/plant height + root dry weight/shoot dry weight) × total plant dry weight (Guimarães et al., 2024).

### Photosynthetic pigment and chlorophyll fluorescence analysis

For photosynthetic pigment determination, five seedlings were randomly selected per treatment, the 3rd to 5th fresh leaves were collected, surface-cleaned and cut into small pieces (1–2 mm wide, excluding midribs). Approximately 0.100 g of chopped leaf tissue was accurately weighed (three replicates) and placed in test tubes containing 20 mL 95% ethanol. The samples were incubated in the dark for 24 h, and the absorbance was measured at 470 nm, 649 nm, and 665 nm using a dual-beam spectrophotometer (UV-6100S, Mapada Instruments Co., Ltd., Shanghai, China) (Wellburn, 1994). Chlorophyll and carotenoid contents were calculated using the following equations:


$$\text{Chl a }\left(\text{mg}/\text{L}\right)=13.95\;\times {\text{ A}}_{665}-\;6.88\;\times {\text{ A}}_{649}$$

$$\text{Chl b }\left(\text{mg}/\text{L}\right)=24.96\times {\text{A}}_{649}-7.32\times {\text{A}}_{665}$$

$$\text{Carotenoids }\left(\text{mg}/\text{L}\right)=\left(1000\;\times {\text{ A}}_{470}-2.05\times \text{Chl a}-114.8\times \text{Chl b}\right)/245$$

Total Chl=Chl a+Chl b

Chlorophyll fluorescence parameters were measured using a portable chlorophyll fluorometer (PAM-2500; Walz, Germany) (Baker, 2008). Selected fully expanded functional leaves were fitted with instrument leaf clips and subjected to 30 min of dark adaptation prior to measurement to ensure full relaxation of PSII reaction centers for reliable F_v_/F_m_ determination.

### Determination of chlorophyll metabolite contents

The levels of 5-aminolevulinic acid (ALA), porphobilinogen (PBG), uroporphyrinogen III (Urogen III), and coproporphyrinogen III (Coprogen III) were determined using enzyme-linked immunosorbent assay (ELISA) kits (Shanghai Yuanye Bio-Technology Co., Ltd., China; ALA: TW49512B; PBG: TW421212B; Urogen III: TW42122B; Coprogen III: TW42422B) according to the instructions. Absorbance was measured at 450 nm using a microplate reader, and the concentrations were calculated from the standard curves (Nguyen et al., 2023). Protoporphyrin IX (Proto IX), Mg-protoporphyrin IX (Mg-Proto IX), and protochlorophyllide (Pchlide) were extracted and measured as described by Hodgins and Van Huystee (Hodgins and Van Huystee, 1986). Three independent biological replicates were performed for the analysis of each substance, with each replicate consisting of pooled leaf samples collected from five seedlings. The leaf tissue was ground in liquid nitrogen and extracted using 10 mL of 80% alkaline acetone. The extract was centrifuged at 10,000 × g for 15 min at 4 °C, and the absorbance of the supernatant was measured at 575 nm, 590 nm, and 628 nm.

### Plant mineral element analysis

Mineral element analysis was conducted with three independent biological replicates, and each replicate contained dried leaf samples pooled from five seedlings. Dried leaf samples were ground using a ball mill, passed through a 0.5-mm sieve, and stored in sealed bags at room temperature for subsequent mineral analysis. The TN was determined using Kjeldahl digestion with H_2_SO_4_-H_2_O_2_ (Kalra, 1997). Magnesium (Mg) and iron (Fe) concentrations were determined using atomic absorption spectrophotometry after digestion with HNO_3_-HClO_4_ (Majkowska-Gadomska et al., 2024).

### Statistical analysis

All data were presented as the mean ± standard error (n = 3). Statistical analyses were performed using SPSS software (version 26.0; IBM Corp., Armonk, NY, USA). Figures were prepared using Microsoft Excel 365. Differences among treatments were analyzed using one-way analysis of variance (ANOVA), followed by Duncan’s multiple range test at *p* < 0.05.

## Results

### Vermicompost application improves growing substrate physicochemical properties and enhances nutrient availability

**Figure 1:**
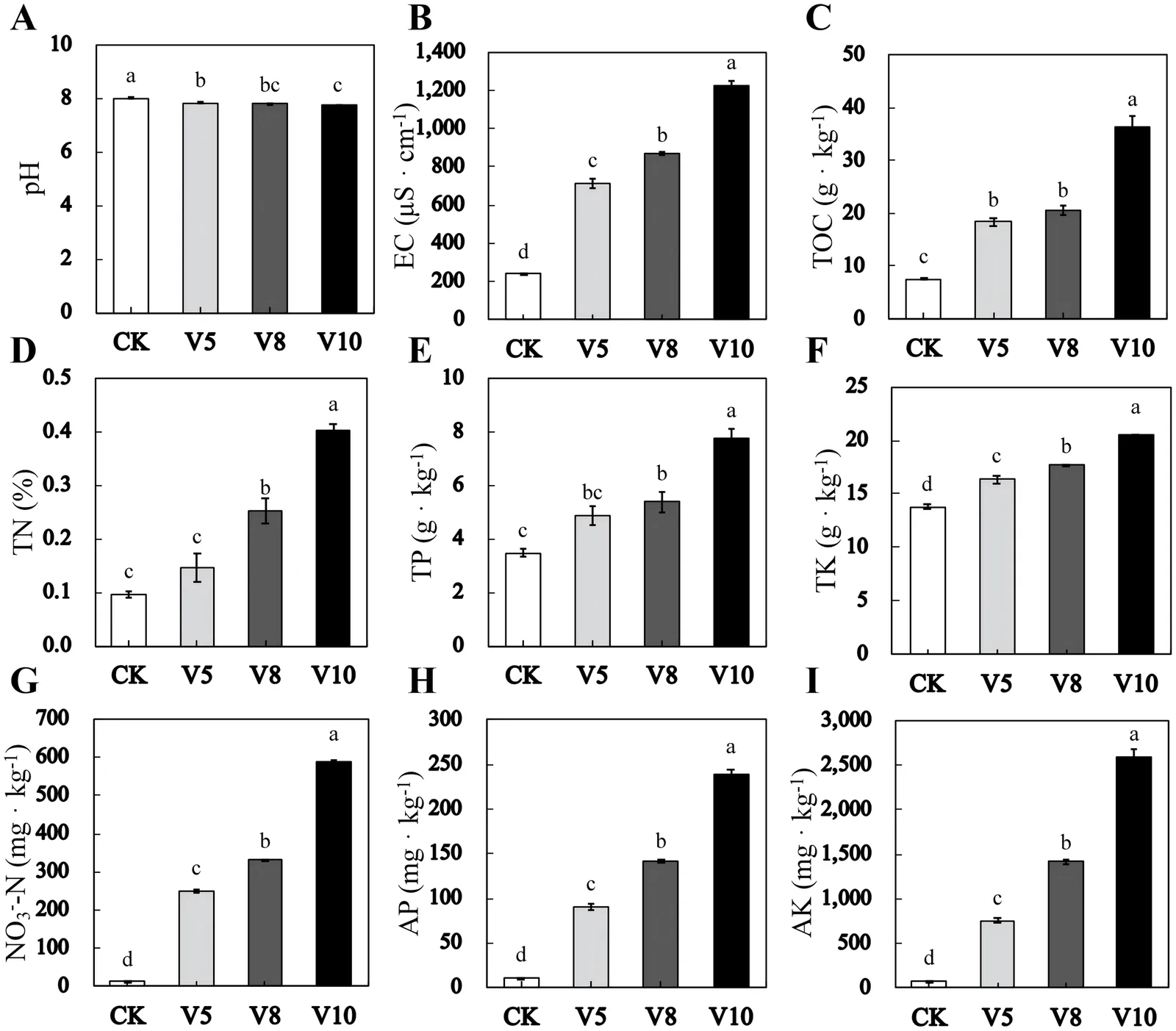
Effects of different proportions of vermicompost on the chemical characteristics of growing substrate **(A)** Potential of Hydrogen, **(B)** Electrical Conductivity, **(C)** growing substrate Organic Carbon, **(D)** Total Nitrogen, **(E)** Total Phosphorus, **(F)** Total Potassium, **(G)** Nitrate Nitrogen, **(H)** Available Phosphorus, **(I)** Available Potassium were determined after 20 days of treatment. CK: 0 percent vermicompost and 100 percent growing substrate; V5: 50 percent vermicompost and 50 percent growing substrate; V8: 80 percent vermicompost and 20 percent growing substrate; V10: 100 percent vermicompost and 0 percent growing substrate. Data are means ± SE (n = 3). Different letters indicate significant differences (*p* < 0.05, Duncan’s test).

To evaluate the basic effects of vermicompost application, we first examined the changes in fundamental growing substrate physicochemical properties, including pH, electrical conductivity (EC), and total organic carbon (TOC) (**Figure 1**). Growing substrate pH progressively decreased with increasing vermicompost ratios, dropping from 8.02 in the control (CK) to 7.83, 7.81, and 7.78 in the V5, V8, and V10 treatments, which represented significant reductions of 2.37%, 2.62%, and 3.03%, respectively. In contrast, all vermicompost treatments substantially elevated growing substrate EC. From a basal level of 238.67 μS cm^-1^ in the CK, EC values rose to 713.67 μS cm^-1^ and 869.33 μS cm^-1^ in the V5 and V8 treatments (1.99- and 2.64-fold increases), eventually peaking at 1222.00 μS cm^-1^ under the V10 treatment (a 4.12-fold increase). Similarly, TOC content was significantly enhanced, increasing from 7.51 g kg^-1^ in the CK to 36.28 g kg^-1^ in the V10 treatment (a 3.83-fold increase). The V5 and V8 treatments also showed significant TOC increases of 1.45 and 1.74 times compared to the control, respectively.

Total growing substrate nutrient contents were also significantly enhanced by vermicompost application, particularly at higher incorporation ratios (**Figure 1**). Rather than a uniform increase across all nutrients, total nitrogen (TN) showed the most notable response. In the V8 treatment, TN reached 0.25%, representing a 1.65-fold increase over the initial CK level of 0.10%. Concurrently, total phosphorus (TP) and total potassium (TK) in this treatment rose to 5.40 g kg^-1^ and 17.66 g kg^-1^ (increases of 0.55 and 0.28 times, respectively). The V10 treatment drove these metrics even higher; TN peaked at 0.40% (a 3.21-fold increase), while TP and TK reached 7.75 g kg^-1^ and 20.47 g kg^-1^ (1.23 and 0.48 times higher than the control, respectively).

Furthermore, the pool of available growing substrate nutrients expanded dramatically across all vermicompost treatments (**Figure 1**). Compared to the control plots, the V5 treatment induced a rapid accumulation of available nutrients. Specifically, nitrate nitrogen (NO_3_^-^-N) surged from 12.16 mg kg^-1^ in the CK to 248.43 mg kg^-1^ in V5 (a 19.42-fold increase). Similar robust trends were observed for available phosphorus (AP) and available potassium (AK), which climbed to 90.51 mg kg^-1^ and 759.28 mg kg^-1^ (7.66 and 8.33 times their respective CK levels of 10.45 mg kg^-1^ and 81.37 mg kg^-1^). This enrichment effect was most pronounced in the V10 treatment, where NO_3_^-^-N exhibited a striking 47.31-fold increase to reach 587.60 mg kg^-1^. AP and AK contents also peaked at this highest application rate, attaining values of 239.04 mg kg^-1^ and 2592.81 mg kg^-1^—corresponding to dramatic increases of 21.86 and 30.87 times relative to the control.

### Vermicompost promotes apple seedling growth and quality

**Figure 2:**
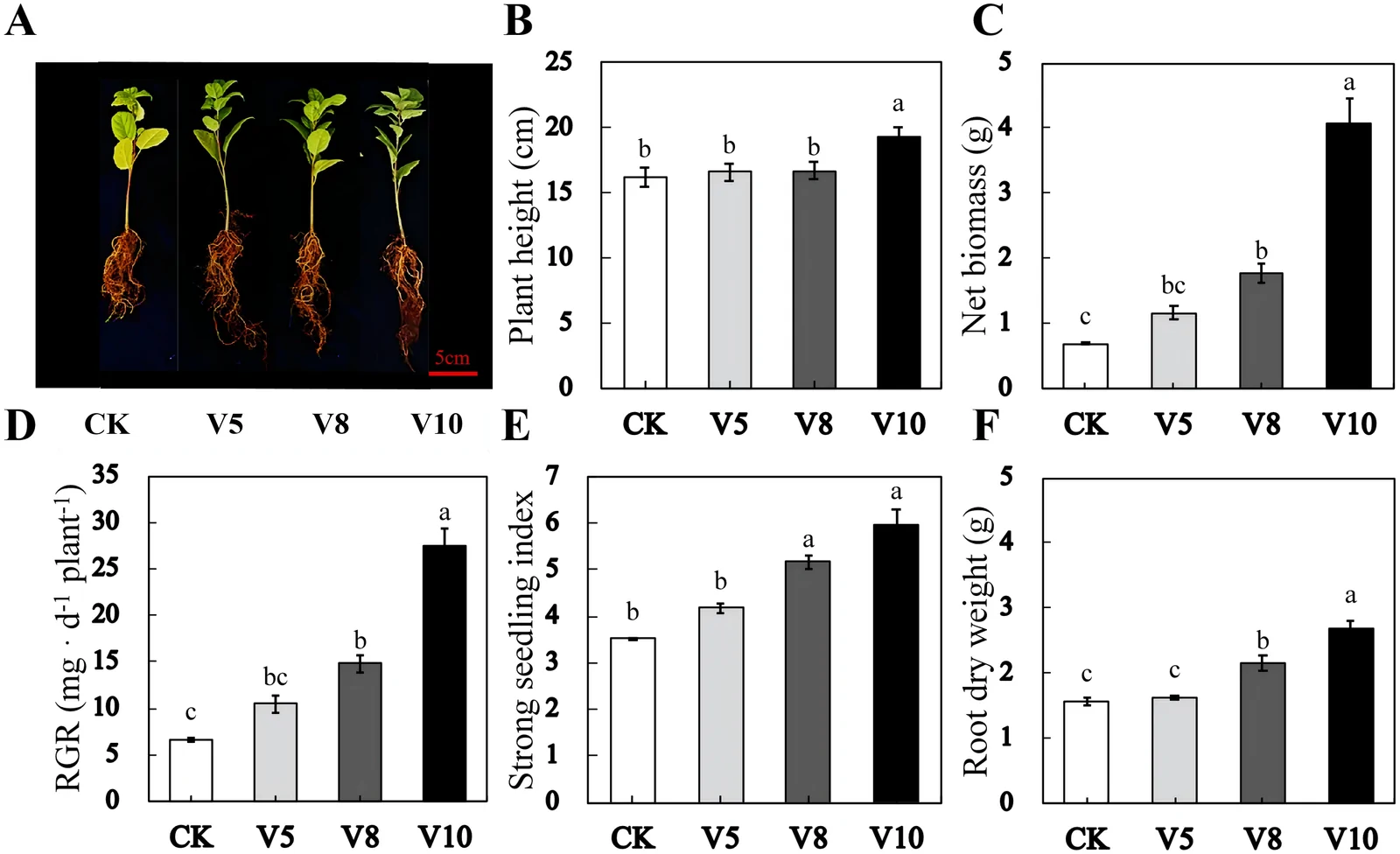
Effects of different proportions of vermicompost on the growth of apple seedlings. **(A)** Seedling morphology, **(B)** Plant height, **(C)** Net biomass, **(D)** relative growth rate, **(E)** Strong seedling index, **(F)** Root dry weight were determined after 20 days of treatment. CK: 0 percent vermicompost and 100 percent growing substrate; V5: 50 percent vermicompost and 50 percent growing substrate; V8: 80 percent vermicompost and 20 percent growing substrate; V10: 100 percent vermicompost and 0 percent growing substrate. Data are means ± SE (n = 3). Different letters indicate significant differences (*p* < 0.05, Duncan’s test).

To determine whether the vermicompost-induced growing substrate improvements translated into biological benefits, we further evaluated the growth performance of apple seedlings. Morphological observation of the seedlings after 20 days revealed that vermicompost-treated plants generally appeared healthier and taller, with visually darker green leaves relative to the control (**Figure 2A**). Analysis of plant height confirmed this promotional effect. While the control (CK) plants reached an average height of 16.20 cm, the V10 treatment yielded significantly taller seedlings at 19.30 cm, representing a 19.11% increase over the control (**Figure 2B**). In contrast, plant heights for the intermediate V5 and V8 treatments did not differ significantly from CK (**Figure 2B**).

Consistent with the morphological results, vermicompost application profoundly enhanced biomass accumulation and growth rates. Compared to the baseline CK values of 0.69 g for net biomass and 6.59 mg · d^-1^ plant^-1^ for relative growth rate (RGR), high vermicompost proportions (V8 and V10) triggered substantial gains (**Figures 2C, D**). The V10 treatment exhibited the maximum values for both metrics, reaching 4.06 g and 27.51 mg · d^-1^ plant^-1^. These represent striking increases of 488.88% and 317.29% over the control, respectively. Similarly, the V8 treatment elevated net biomass to 1.77 g (a 156.52% increase) and RGR to 14.83 mg · d^-1^ plant^-1^ (a 124.93% increase). No significant enhancements in biomass or RGR were observed for the lowest V5 application proportion, which maintained levels comparable to CK.

The overall quality and root development of the seedlings were also dramatically improved by vermicompost addition. From a baseline Strong Seedling Index of 3.51 and a root dry weight of 1.57 g, high vermicompost proportions drove significant enhancements (**Figures 2E, F**). Maximum growth was achieved under the V10 treatment, which elevated the Strong Seedling Index to 5.96 (up 69.55%) and root dry weight to 2.69 g (up 70.94%). The V8 treatment also showed marked improvements, raising the Strong Seedling Index to 5.17 (up 47.06%) and root dry weight to 2.16 g (up 37.08%). In contrast, while the V5 treatment significantly increased the Strong Seedling Index to 4.17, its impact on root dry weight remained statistically similar to the CK group (**Figure 2E**).

### Vermicompost enhances the light energy utilization and photosynthetic efficiency of apple seedlings

**Figure 3:**
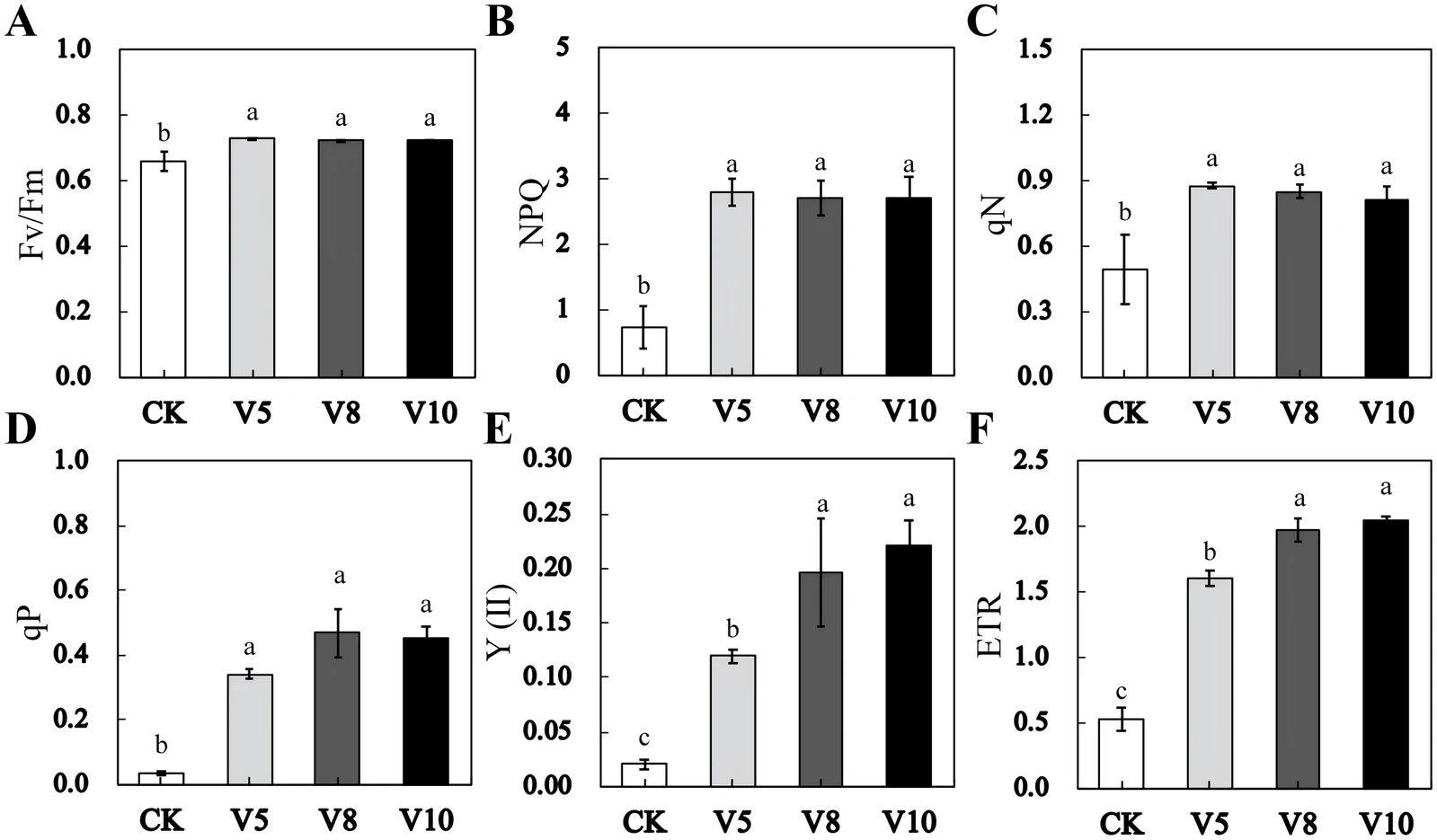
Effects of different proportions of vermicompost on the chlorophyll fluorescence parameters. **(A)** Maximum quantum yield of PSII (F_v_/F_m_):, **(B)** Actual quantum yield of PSII in the light [Y(II)], **(C)** Non-photochemical quenching (NPQ), **(D)** Electron transport rate (ETR), **(E)** Non-photochemical quenching coefficient (qN), **(F)** Photochemical quenching coefficient (qP) were determined after 20 days of treatment. CK: 0 percent vermicompost and 100 percent growing substrate; V5: 50 percent vermicompost and 50 percent growing substrate; V8: 80 percent vermicompost and 20 percent growing substrate; V10: 100 percent vermicompost and 0 percent growing substrate. Data are means ± SE (n = 3). Different letters indicate significant differences (*p* < 0.05, Duncan’s test).

To determine the effect of vermicompost on the light energy utilization of apple seedlings, chlorophyll fluorescence parameters were measured (**Figure 3**). Compared to the control (CK), the addition of vermicompost significantly increased the maximum quantum yield of PSII (F_v_/F_m_), non-photochemical quenching (NPQ), non-photochemical quenching coefficient (qN), and photochemical quenching coefficient (qP) across all treatments (V5, V8, V10), with no significant differences observed among the vermicompost groups themselves (**Figures 3A–D**). Moreover, the actual quantum yield of PSII [Y(II)] and electron transport rate (ETR) exhibited an upward trend with increasing vermicompost proportions. The V10 treatment resulted in the highest Y(II) and ETR values, significantly outperforming the CK group (**Figures 3E, F**). These findings clearly indicate that vermicompost treatments enhance the photosynthetic efficiency of apple seedlings.

### Vermicompost promotes photosynthetic pigment accumulation without altering compositional balance

**Figure 4:**
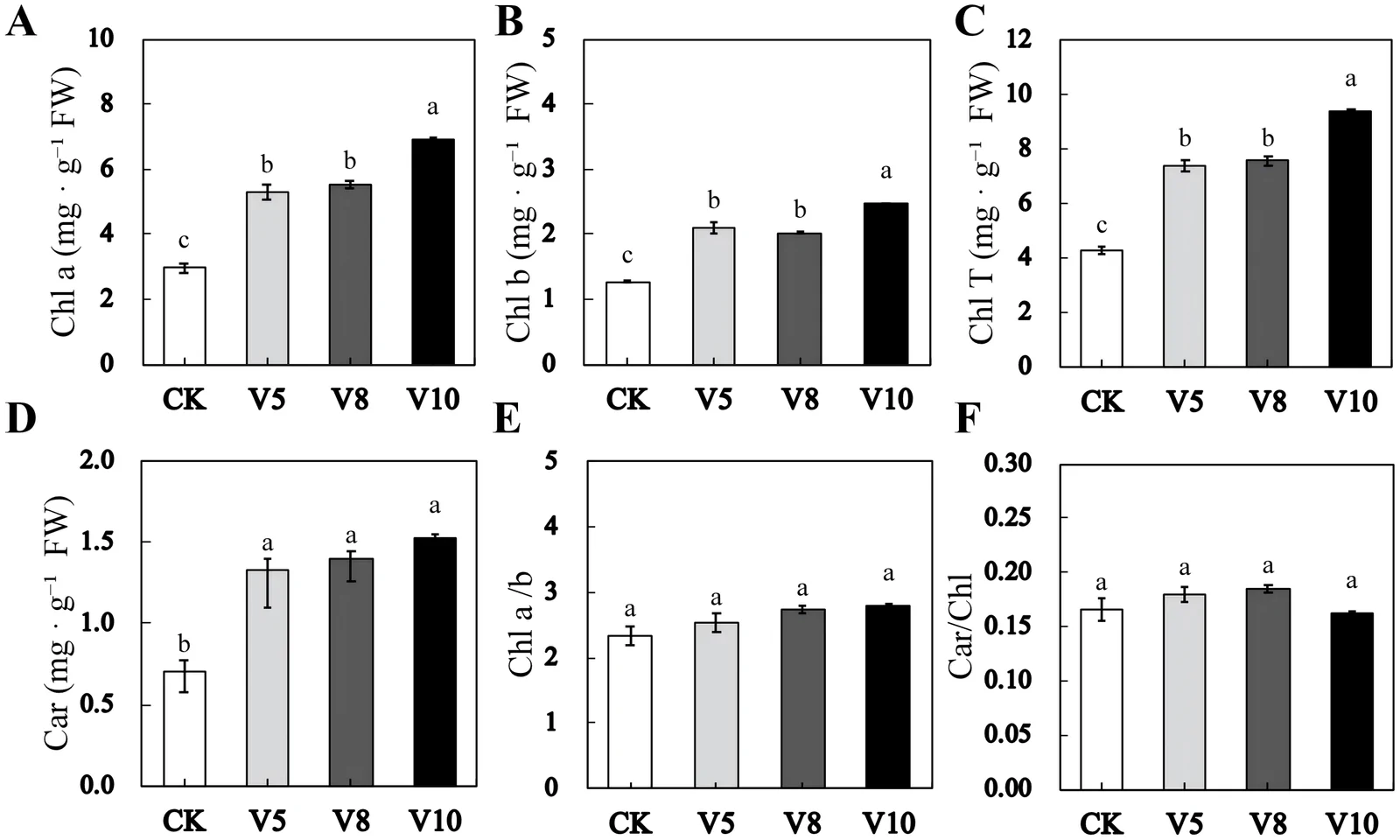
Effects of different proportions of vermicompost on the photosynthetic pigment content in leaves. **(A)** Chlorophyll a content, **(B)** Chlorophyll b content, **(C)** Carotenoid content, **(D)** Total Chlorophyll content, **(E)** Chlorophyll a/b, **(F)** Carotenoid/Chlorophyll were determined after 20 days of treatment. CK: 0 percent vermicompost and 100 percent growing substrate; V5: 50 percent vermicompost and 50 percent growing substrate; V8: 80 percent vermicompost and 20 percent growing substrate; V10: 100 percent vermicompost and 0 percent growing substrate. Data are means ± SE (n = 3). Different letters indicate significant differences (*p* < 0.05, Duncan’s test).

To further elucidate the basis of this improved light energy utilization, we analyzed the photosynthetic pigment contents in the leaves (**Figure 4**). Consistent with the fluorescence data, the V10 treatment yielded the highest concentrations of chlorophyll a, chlorophyll b, total chlorophyll, and carotenoids. Specifically, compared with CK, the V5, V8, and V10 treatments significantly increased chlorophyll a content by 78.01%, 86.11%, and 132.18%, respectively; chlorophyll b by 64.74%, 58.81%, and 94.00%; and total chlorophyll by 74.03%, 77.92%, and 120.73%. Similarly, carotenoid levels increased significantly by 87.45% to 115.25% in the vermicompost-treated groups relative to CK, although no significant differences were detected among the V5, V8, and V10 treatments. Despite these substantial increases in absolute pigment concentrations, the Chl a/b and Car/Chl ratios exhibited no significant differences across any of the experimental groups.

### Vermicompost enhances the accumulation of key mineral nutrients for chlorophyll synthesis

**Figure 5:**
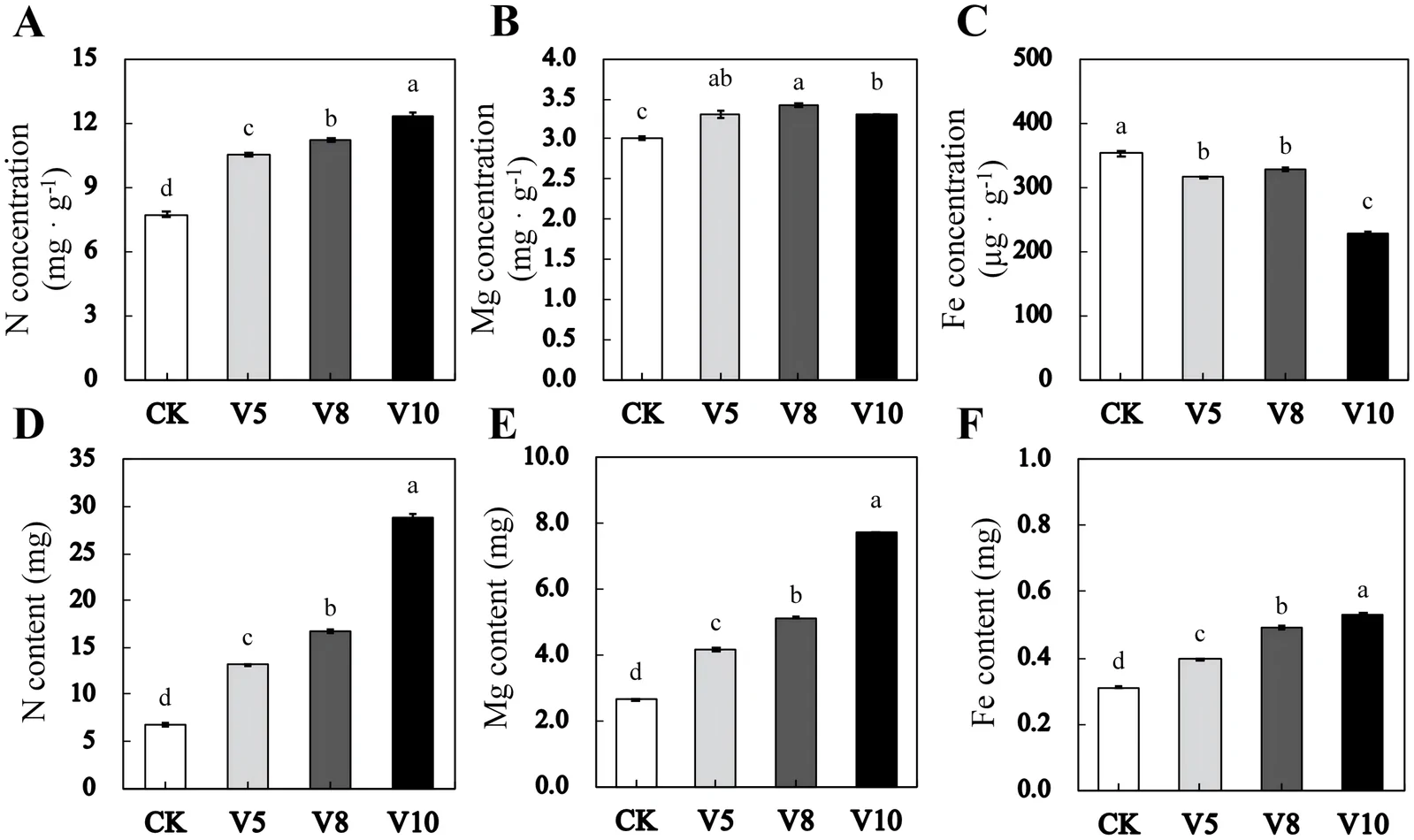
Effects of different proportions of vermicompost on the mineral nutrient content of leaves. **(A)** Nitrogen concentration, **(B)** Magnesium concentration, **(C)** Iron concentration, **(D)** Nitrogen content, **(E)** Magnesium content, **(F)** Iron content were determined after 20 days of treatment. CK: 0 percent vermicompost and 100 percent growing substrate; V5: 50 percent vermicompost and 50 percent growing substrate; V8: 80 percent vermicompost and 20 percent growing substrate; V10: 100 percent vermicompost and 0 percent growing substrate. Data are means ± SE (n = 3). Different letters indicate significant differences (*p* < 0.05, Duncan’s test).

Following the observation of increased chlorophyll levels, we analyzed the impact of vermicompost on the accumulation of nitrogen (N), magnesium (Mg), and iron (Fe)—essential mineral elements involved in chlorophyll synthesis (**Figure 5**). The mineral concentrations in apple leaves exhibited divergent responses to the treatments (**Figures 5A–C**). Leaf N concentration increased progressively with higher vermicompost proportions, peaking in the V10 treatment at 12.34 mg g^-1^ (a 59.95% increase over CK). Mg concentration was also significantly elevated by vermicompost, reaching its maximum under the V8 treatment. In contrast, leaf Fe concentration decreased significantly across all vermicompost-treated groups compared to the control (CK), with V10 exhibiting the lowest concentration.

Despite the varying trends in relative concentration, the absolute mineral content of all three elements (N, Mg, and Fe) displayed a consistent and significant upward trend corresponding with increased vermicompost application (**Figures 5D–F**). Compared to the CK group, the total foliar contents of N, Mg, and Fe peaked under the V10 treatment at 28.79 mg, 7.70 mg, and 0.53 mg, representing dramatic increases of 325.68%, 191.45%, and 70.87%, respectively. This discrepancy suggests a dilution effect due to rapid biomass accumulation, which will be addressed in the discussion

### Vermicompost promotes chlorophyll biosynthesis rather than inhibiting degradation

**Figure 6:**
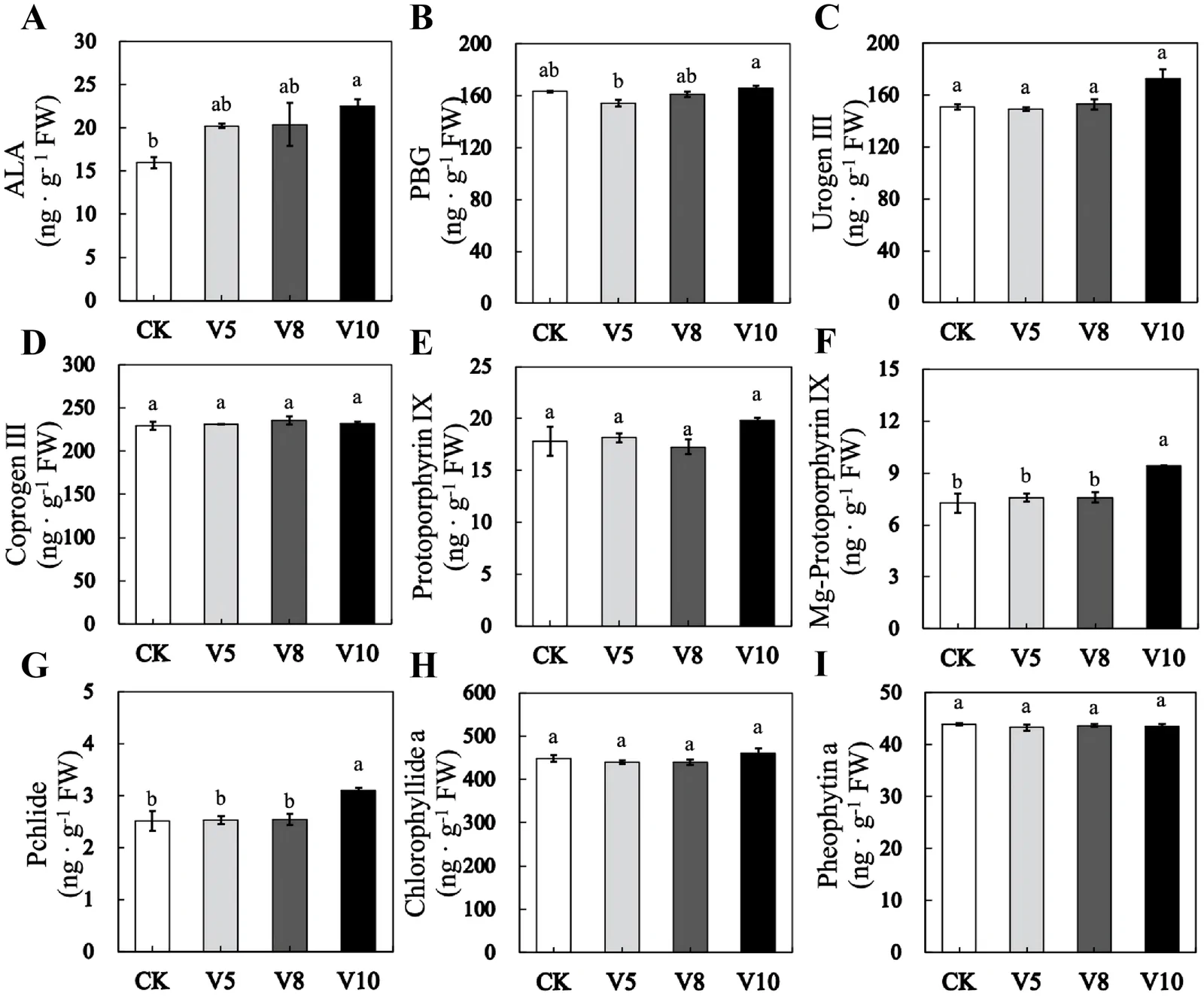
Effects of different proportions of vermicompost on chlorophyll metabolism. **(A)** Aminolevulinic acid, **(B)** Porphobilinogen, **(C)** Uroporphyrinogen III, **(D)** Coproporphyrinogen III, **(E)** Protoporphyrin IX, **(F)** Magnesium-protoporphyrin IX, **(G)** Protochlorophyllide, **(H)** Chlorophyllide a, **(I)** Pheophytin a were determined after 20 days of treatment. CK: 0 percent vermicompost and 100 percent growing substrate; V5: 50 percent vermicompost and 50 percent growing substrate; V8: 80 percent vermicompost and 20 percent growing substrate; V10: 100 percent vermicompost and 0 percent growing substrate. Data are means ± SE (n = 3). Different letters indicate significant differences (*p* < 0.05, Duncan’s test).

To further elucidate the metabolic mechanisms underlying the elevated chlorophyll content, we profiled the intermediate precursors and key degradation products in the chlorophyll metabolic pathway (**Figure 6**). Significant variations were observed among the treatments for several precursors in the chlorophyll biosynthetic pathway. The ALA content was significantly higher in all vermicompost-treated seedlings than in the CK. Specifically, ALA contents reached 20.22 ng g^-1^, 20.39 ng g^-1^, and 22.47 ng g^-1^ in the V5, V8, and V10 treatments, representing increases of 26.54%, 27.62%, and 40.64%, respectively (**Figure 6A**). For PBG, although the V10 treatment showed the numerically highest content 165.85 ng g^-1^, it was only significantly greater than the V5 treatment 154.40 ng g^-1^, with no statistically significant differences compared to CK or V8 (**Figure 6B**). Similarly, the contents of Mg-Protoporphyrin IX and Pchlide were significantly elevated only under the V10 treatment, peaking at 9.41 ng g^-1^ and 3.10 ng g^-1^. These represented increases of 29.81% and 22.85% compared to their respective CK values 7.25 ng g^-1^ and 2.52 ng g^-1^ (**Figures 6F, G**).

In contrast, other key intermediate precursors, such as Urogen III, Coprogen III, and protoporphyrin IX, showed no statistically significant differences across the different treatments (**Figures 6C–E**). Regarding chlorophyll degradation, the contents of the degradation products pheophytin a and chlorophyllide a remained consistent, with no significant differences observed among all treatments (**Figures 6H, I**).

## Discussion

The primary objective of this study was to elucidate the regulatory mechanisms of vermicompost on the interconnected growing substrate –plant system, specifically evaluating its impact on root-zone microenvironments, photosynthetic efficiency, and chlorophyll metabolism in apple seedlings. Our findings demonstrate that vermicompost acts as a highly effective organic growing substrate amendment, profoundly improving growing substrate physicochemical properties and nutrient availability and thereby stimulating vigorous shoot and root growth.

### Promotion of apple seedling growth and root development under vermicompost amendment

As expected, vermicompost application markedly modified background growing substrate parameters, decreasing pH and substantially increasing both total and available N, P, and K, particularly at the highest rate (V10). Similar increases in nutrient availability have been widely documented as the foundational mechanism by which vermicompost enhances soil fertility (Lim et al., 2015; Sharma et al., 2025). In our study, rather than treating these growing substrate modifications as an independent outcome, we view them as the critical environmental parameters that directly interpret the subsequent biological responses of the apple seedlings. Specifically, this optimized growing substrate microenvironment and massive nutrient enrichment directly translated into superior morphological development. Vermicompost-treated plants were taller, had greater biomass, and developed more extensive root systems, with V8 and V10 producing the largest gains in Relative Growth Rate and Strong Seedling Index. Enhanced vegetative growth under vermicompost application has also been observed inlettuce, rice, tomato, chickpea, fenugreek and watercress, where modest vermicompost rates significantly increased shoot biomass, root traits, and overall plant vigor (Baghbani-Arani et al., 2017; Ruan et al., 2021; Mishra et al., 2023; Toor et al., 2024; Belay et al., 2025; Mohebbi et al., 2024);. For example, nursery raising of fragrant rice with vermicompost increased plant height, stem diameter, dry weight, and root length by 11–65% relative to the control (Ruan et al., 2021), while lettuce grown with 2–4% vermicompost showed up to 56% yield increases accompanied by higher soil N and enzyme activities (Toor et al., 2024). Compared with these studies, the biomass response of apple seedlings, up to nearly five-fold in V10, suggests that woody perennials in containerized nursery conditions may exhibit particularly high sensitivity to vermicompost-driven nutrient and structural improvements.

### Enhanced photosynthetic performance and photoprotection under vermicompost amendment

This robust vegetative growth was fundamentally driven by enhanced light energy utilization and photosynthetic capacity. In our study, vermicompost significantly increased F_v_/F_m_, Y(II), and ETR, especially in V8 and V10, while NPQ and qN also rose, indicating both improved photochemical efficiency and strengthened photoprotection. Similar improvements in chlorophyll fluorescence parameters under vermicompost have been reported in several crops. Vermicompost application increased F_v_/F_m_, net photosynthetic rate, and related fluorescence traits in tomato, with vermicompost outperforming biochar at equivalent application rates (Liu et al., 2022). In chickpea, vermicompost enhanced F_v_/F_m_ and net photosynthetic rate under well-watered conditions, though benefits diminished under severe drought (Hosseinzadeh et al., 2016). In fenugreek, vermicompost mitigated water-deficit-induced reductions in F_v_/F_m_ and chlorophyll content, partially alleviating photoinhibition (Baghbani-Arani et al., 2017). The magnitude and consistency of the fluorescence improvements observed in our apple seedlings across all higher vermicompost levels align with these findings, but also indicate that under non-stress nursery conditions vermicompost can elevate PSII performance to an unusually high level, accompanied by strong thermal dissipation capacity.

The elevated light energy capture efficiency in our study was tightly coupled to massive accumulations of photosynthetic pigments. The V10 treatment produced the highest concentrations of chlorophyll a, chlorophyll b, carotenoids, and total chlorophyll without altering Chl a/b or Car/Chl ratios. In other crop systems, vermicompost similarly increases chlorophyll content. Vermicompost nursery matrices for fragrant rice significantly raised chlorophyll a, chlorophyll b, carotenoids, and total chlorophyll compared with controls (Ruan et al., 2021), while tomato and chickpea treated with vermicompost showed higher chlorophyll levels and improved gas exchange (Mishra et al., 2023; Belay et al., 2025). Our results are in agreement with this body of work but add the observation that pigment stoichiometry remains stable even when total pigment levels more than double, suggesting that vermicompost stimulates an expansion of the light-harvesting antenna rather than a restructuring of pigment–protein complexes.

### Vermicompost-regulated mineral nutrient balance supports photosynthetic function

The synthesis of these tetrapyrrole pigments requires an uninterrupted supply of specific mineral elements. In our apple seedlings, foliar N and Mg concentrations rose significantly with increasing vermicompost rate, while Fe concentration declined but total foliar N, Mg, and Fe contents all increased markedly, peaking in V10. The importance of N and Mg in sustaining chlorophyll and photosynthesis has been emphasized in several studies. In citrus, Mg deficiency led to interveinal chlorosis, reduced F_v_/F_m_, and pronounced impairment of photosynthetic electron transport, highlighting Mg’s central role in chlorophyll structure and thylakoid function (Ye et al., 2019). Likewise, Mg supply affected mineral balance, chlorophyll fluorescence parameters, and nutrient distribution in sweet orange, underscoring the need for adequate Mg management to maintain photosynthetic performance (Papadakis et al., 2023). Our observation that vermicompost-enhanced Mg supply coincided with higher Mg-protoporphyrin IX levels and improved PSII efficiency in apple seedlings aligns well with these findings, suggesting that vermicompost alleviates potential Mg limitations in nursery substrates.

Iron is equally critical for chlorophyll biosynthesis and electron transport. Iron deficiency in Areca catechu caused chloroplast degeneration, reduced chlorophyll synthesis, and severe chlorosis, whereas excessive Fe increased chlorophyll content but still depressed growth and photosynthesis (Li et al., 2021). Broader reviews of mineral signaling have highlighted Fe deficiency as a major trigger of chlorosis and impaired photosynthesis, while also emphasizing complex interactions with other nutrients such as P and S (Therby-Vale et al., 2022). In our study, the decline in leaf Fe concentration but rise in total Fe content under high vermicompost rates, together with intense green pigmentation and high F_v_/F_m_, supports a dilution effect rather than true Fe deficiency. This contrasts sharply with the chlorosis and structural damage reported under Fe-deficient conditions (Li et al., 2021; Therby-Vale et al., 2022), indicating that vermicompost can increase overall Fe uptake sufficiently to support pigment synthesis even as rapid biomass accumulation dilutes tissue Fe concentrations. Quantitatively, the ~5.9-fold increase in total plant biomass under the V10 treatment substantially exceeded the ~1.7-fold increase in total foliar Fe content, further corroborating that the reduction in Fe concentration is driven by biomass dilution. Additionally, the slight decrease in substrate pH induced by vermicompost, combined with synergistic effects of associated beneficial microbes, may have enhanced Fe solubility and bioavailability, which contributes to maintaining adequate Fe uptake, pigment synthesis and photosynthetic performance despite the dilution effect (Shekari et al., 2026).

### Modulation of chlorophyll metabolic flux toward biosynthesis by vermicompost

Beyond bulk mineral supply, our targeted metabolite measurements revealed that vermicompost primarily promoted chlorophyll biosynthesis rather than inhibiting degradation. ALA content increased significantly in all vermicompost treatments, with V10 showing an increase of over 40%, and downstream intermediates Mg-protoporphyrin IX and protochlorophyllide were also elevated, whereas other intermediates remained unchanged and degradation products (pheophytin a, chlorophyllide a) were stable. This metabolic pattern closely parallels observations in studies where exogenous ALA was used to enhance chlorophyll synthesis under stress. In cucumber, ALA application under salt stress increased the expression of genes in the chlorophyll branch (GluTR, Mg-chelatase, POR), raised levels of Proto IX, Mg-Proto IX, Pchlide, and chlorophyll, and restored photosynthetic performance (Wu et al., 2018). In Kentucky bluegrass and Brassica napus, ALA increased endogenous ALA, Mg-Proto IX, Pchlide, and chlorophyll content while improving PSII efficiency, ETR, and net photosynthesis under osmotic or salt stress (Niu and Ma, 2018; Xiong et al., 2018). Recent work in wheat further showed that ALA promotes chlorophyll precursor synthesis by enhancing activities of key enzymes (e.g., 5-amino-ketovalerate dehydratase, uroporphyrinogen III synthase), whereas dihydroporphyrin iron chelate primarily reduces chlorophyllase activity and slows degradation (Hu et al., 2024). Our findings are consistent with this ALA-centered model, but are novel in demonstrating that an organic growing substrate amendment, rather than direct ALA application, can upregulate ALA and specific downstream precursors in the chlorophyll pathway under non-stress conditions in a woody perennial.

The role of ALA as a regulator of porphyrin flux between chlorophyll and heme branches has been further clarified in the chimeric leaves of Ananas comosus var. bracteatus, where moderate increases in ALA redirected porphyrin metabolism toward heme and inhibited chlorophyll synthesis, whereas higher ALA levels favored chlorophyll biosynthesis (Adjei et al., 2023). This bidirectional regulation underscores the need for balanced ALA production to maintain porphyrin homeostasis. In our study, the coordinated increases in ALA, Mg-protoporphyrin IX, protochlorophyllide, and total chlorophyll, together with stable chlorophyll degradation products, suggest that vermicompost promotes a level of ALA production that favors chlorophyll branch flux without inducing heme overaccumulation or metabolic imbalance. This contrasts with stress-induced shifts toward heme reported in some salt-stressed systems lacking ALA supplementation (Wu et al., 2018; Xiong et al., 2018), and supports the interpretation that vermicompost encourages a controlled, biosynthesis-driven expansion of the chlorophyll pool. Notably, these findings demonstrate that an organic soil amendment per se can recapitulate the chlorophyll-promoting effects typically achieved via exogenous ALA application under stress, extending this regulatory pathway to non-stress nursery conditions in woody perennials.

## Conclusion

This study demonstrates that vermicompost incorporation profoundly enhances apple (G935) seedling growth. The 100% vermicompost treatment (V10) maximized growing substrate nutrient availability and optimized physicochemical properties, driving significant increases in seedling biomass and root development. This morphological improvement was underpinned by enhanced light energy capture and photosystem II efficiency. Crucially, metabolic profiling revealed that vermicompost upregulates photosynthetic capacity by stimulating chlorophyll biosynthesis (increasing ALA, Mg-Proto IX, and Pchlide) and improving essential mineral uptake (N, Mg, Fe), without altering pigment degradation. Notably, the 50% vermicompost treatment (V5) generally produced intermediate or non-significant effects across most traits, indicating that incorporation ratios of ≥80% are required to achieve maximal benefits. Ultimately, high-ratio vermicompost provides a highly effective, sustainable substrate management strategy for optimizing apple nursery production. While 100% vermicompost yields maximal physiological benefits, large-scale application must consider material availability and cost. Therefore, in actual cultivation, vermicompost can be blended with low-cost conventional matrix materials to cut production costs and facilitate large-scale promotion in commercial nurseries.

## Data Availability

The raw data supporting the conclusions of this article will be made available by the authors, without undue reservation.
